# Age-specific diabetes risk by the number of metabolic syndrome components: a Korean nationwide cohort study

**DOI:** 10.1186/s13098-019-0509-8

**Published:** 2019-12-27

**Authors:** Min-Kyung Lee, Kyungdo Han, Hyuk-Sang Kwon

**Affiliations:** 1 Division of Endocrinology and Metabolism, Department of Internal Medicine, Myongji Hospital, Hanyang University College of Medicine, Goyang, Gyeonggi-do Republic of Korea; 2 Department of Medical Statistics, College of Medicine, The Catholic University of Korea, Seoul, Republic of Korea; 3 Division of Endocrinology and Metabolism, Department of Internal Medicine, Yeouido St. Mary’s Hospital, College of Medicine, The Catholic University of Korea, Seoul, Republic of Korea

**Keywords:** Metabolic syndrome, Risk factors, Diabetes mellitus, Type 2

## Abstract

**Background:**

Metabolic syndrome is associated with an increased risk of diabetes. This study investigated the associations between the number of metabolic syndrome components and diabetes risk by age, sex and BMI.

**Methods:**

Data for 19,475,643 participants ≥ 20 years old with no history of diabetes were obtained between 2009 and 2012 and were accessed using the South Korean National Health Insurance Service. Metabolic syndrome was defined according to the modified criteria of the National Cholesterol Education Program Adult Treatment Panel III. We assessed the risk of diabetes according to the number of metabolic syndrome components after stratifying the study participants into groups by age (20–39, 46–64, ≥ 65 years), sex, and BMI (below or above 25).

**Results:**

During an average of 5.13 years of follow-up, the incidence rates of diabetes increased with the number of metabolic syndrome components. Age and BMI gradually increased with the number of metabolic syndrome components. The multivariable-adjusted hazard ratios (HRs) for incident diabetes were 1.401, 1.862, 2.47, 3.164 and 4.501 for participants with one through five components, respectively, compared with those without metabolic syndrome components. The risk of diabetes was 1.79-, 2.18-, and 3.05-times higher for participants ≥ 65 years; 2.57-, 3.45-, and 5.18-times higher for participants 40–64 years; and 2.55-, 3.89-, and 6.31-times higher for participants 20–39 years of age with three through five components, respectively, compared to those with no components. There was no difference in the risk of diabetes between men and women. The HRs were 5.63 for participants with a BMI ≥ 25 and 3.98 for those with a BMI < 25 among individuals with five components.

**Conclusions:**

The risk of diabetes was more strongly associated with the number of metabolic syndrome components among younger adults. In addition, the risk of diabetes across the number of metabolic syndrome components was greater in participants with a BMI ≥ 25.

## Background

Metabolic syndrome is a general term given to a clustering of hyperglycaemia, obesity, dyslipidaemia and hypertension [1] and is known to increase the risk for type 2 diabetes and cardiovascular disease [2, 3]. The prevalence of metabolic syndrome is increasing worldwide and this trend has also been observed in Korea [4]. The desired clinical response to metabolic syndrome is improved health outcomes through comprehensive management of the core components of metabolic syndrome [5].

Diabetes risk has been shown to increase with the number of metabolic syndrome components [6–8]. The estimated prevalence of metabolic syndrome differs by age, sex, and ethnicity because variations exist in the frequencies of metabolic components [9–12]. Therefore, the risk of diabetes according to the number of metabolic syndrome components could be different by age, sex, and obesity status. Few studies, however, have analysed diabetes risk in relation to the number of metabolic syndrome components. In addition, the prevalence of metabolic syndrome components increases with age, but the increase in young adults is marked [13]. The presence of metabolic syndrome could represent a lifetime of increased diabetes risk [14]. Moreover, the early identification of metabolic syndrome components could lead to targeted interventions to prevent the development of the syndrome, and thus reduce diabetes risk in later life. Here, we focused on age-specific diabetes risk as a function of the number of metabolic syndrome components to effectively predict the development of diabetes.

In the present large-scale study of a Korean cohort, we investigated the association of the number of metabolic syndrome components at baseline with the development of diabetes over a 5-year period. We also prospectively evaluated the risk of diabetes within particular subgroups based on combinations of age, sex, and obesity status.

## Methods

### Study subjects

We used the database provided by the South Korean National Health Insurance Service (NHIS), a population-based cohort including nearly all South Korean citizens [15]. The database contains all inpatient and outpatient medical claims data including personal information, prescription drugs, diagnostic and treatment codes, and primary and additional diagnostic codes. This study was approved by the NHIS inquiry commission and adhered to the tenets of the Declaration of Helsinki for biomedical research. Since 2015, the South Korean NHIS has released a nationally representative dataset that is open to all researchers whose study protocols are approved by an official review committee. Informed consent was waived by the Institutional Review Board of The Catholic University of Korea (No. SC18ZESI0047) because the national insurance claim data were deidentified for the analysis.

From this cohort, the data collected from 23,317,567 participants over 20 years old between January 2009 and December 2012 were extracted. We excluded 59,805 participants with data missing for at least one variable, as well as 3,782,119 patients with type 2 diabetes. Ultimately, the final study population consisted of 19,475,643 people who had at least one reexamination over 5 years and for whom values for all metabolic syndrome components were measured at baseline. For each participant, the primary outcome between January 1, 2013 and December 31, 2017 was type 2 diabetes, and the number of person-years of follow-up was determined.

### Definition of metabolic syndrome and diagnosis of type 2 diabetes

According to the revised National Cholesterol Education Program Adult Treatment Panel III (NCEP ATP III) criteria [16, 17], metabolic syndrome was diagnosed when three or more of the following five criteria were met: (1) abdominal obesity (waist circumference [WC] ≥ 90 cm for men or 85 cm for women) [18]; (2) elevated triglycerides (fasting triglycerides ≥ 150 mg/dl) or on drug treatment for elevated triglycerides at baseline; (3) reduced HDL cholesterol (< 40 mg/dl for men and < 50 mg/dl for women); (4) elevated blood pressure (BP) (≥ 130 mmHg systolic BP, ≥ 85 mmHg diastolic BP, and/or on antihypertensive drug treatment at baseline, and/or a history of hypertension); and (5) elevated fasting glucose (≥ 100 mg/dl or on drug treatment for elevated glucose).

Type 2 diabetes was present if claims for anti-diabetes drugs were found in the database according to the following criteria: (1) at least one claim per year under the 10th revision of the International Classification of Diseases (ICD)-10 codes E11 (noninsulin-dependent diabetes mellitus), E12 (malnutrition-related diabetes mellitus), E13 (other specified diabetes mellitus), or E14 (unspecified diabetes mellitus), (2) at least one claim per year for anti-diabetes medication prescription, or (3) fasting plasma glucose (FPG) level ≥ 126 mg/dl.

### Measurements and definitions of covariates

Physical examination was performed by measuring height, weight, WC, systolic BP, and diastolic BP according to standardized methods. WC (cm) was measured at the midpoint between the lower border of the rib cage and the iliac crest by trained examiners. BP was measured in triplicate and the mean value of the second and third measurements was used for the analysis. BMI was calculated as weight in kilograms divided by height in square metres (kg/m^2^). Blood samples for the measurement of FPG, HDL cholesterol, and triglyceride levels were obtained in the morning after an overnight fast. Hospitals where these health examinations were performed were certified by the NHIS and subjected to regular quality control.

All participants were required to complete self-administered questionnaires that inquired about smoking and alcohol habits, physical activity, and past medical history. Smoking habits were divided into current smoking and noncurrent smoking. Heavy alcohol consumption was defined as the consumption of ≥ 30 g per day. Regular exercise was defined as performing more than 30 min of moderate physical activity at least five times per week or more than 75 min of strenuous physical activity at least three times per week [19]. Income level was categorized based on the monthly health insurance premiums paid and the population was divided into four income levels. The lowest income population was categorized as the variable.

### Statistical analysis

The baseline characteristics of the participants are presented as mean ± standard deviation (SD) or proportions (%). Geometric means (95% confidence intervals) are used for the distribution that was heavily skewed. 95% CI calculated using Wald method for means. The age- and sex-adjusted incident rates for type 2 diabetes were calculated. Cox proportional hazards regression models were used to estimate hazard ratios (HRs) and 95% CIs for incident diabetes with the adjustment of important risk factors, such as age, sex, alcohol consumption, smoking status, exercise, income, and BMI. The proportional hazard assumption of Cox models was examined by plotting the log minus log survival curves and survival times against cumulative survival. Stratified analyses were performed by age (20–39 vs. 40–64 vs. ≥ 65 years of age), sex (men vs. women), and BMI (below vs. above 25 kg/m^2^), and interactions between subgroups were tested. All statistical tests were two-sided, and P ≤ 0.05 was considered to be statistically significant. All analyses were performed using the Statistical Analysis System statistical software package (version 9.4; SAS Institute, Inc., Cary, NC, USA).

### Availability of data and materials

The authors are unable to share the data analysed in this study because the Korean National Health Insurance Service (NHIS) owns the data. Researchers can request access on the NHIS website (https://nhiss.nhis.or.kr). Details of this process and a provision guide are now available at http://nhiss.nhis.or.kr/bd/ab/bdaba000eng.do.

## Results

### General baseline characteristics

The baseline characteristics of the study population stratified according to the number of metabolic syndrome components are shown in Table 1. Of a total of 19,475,643 participants (9,783,377 men and 9,692,266 women), 6,269,899 (29.7%) had zero components of metabolic syndrome, 5,574,829 (27.4%) had 1, 3,907,472 (20.4%) had 2, 2,389,166 (13.3%) had 3, 1,083,306 (7.1%) had 4, and 250,971 (2.1%) had 5. The mean ages of the participants with zero through five components at baseline were 39.69 ± 12.02, 45.02 ± 13.31, 48.83 ± 13.36, 51.86 ± 13.26, 54.42 ± 13.05 and 56.62 ± 12.56 years, respectively. BMI, WC, triglyceride levels, BP, and FPG gradually increased and HDL cholesterol decreased as the number of metabolic syndrome components increased.

**Table 1:** Baseline characteristics of the study population according to the number of metabolic syndrome components

	Number of components					
	0	1	2	3	4	5
Participants, n	6,269,899	5,574,829	3,907,472	2,389,166	1,083,306	250,971
Age, years	39.69 ± 12.02	45.02 ± 13.31	48.83 ± 13.36	51.86 ± 13.26	54.42 ± 13.05	56.62 ± 12.56
20–39, %	48.87	34.05	24.5	18.1	13.34	9.23
40–64, %	47.81	57.24	62.27	63.68	63.14	62.53
≥ 65, %	3.32	8.72	13.23	18.22	23.51	28.24
Male, %	39.64	52.24	58.27	57.94	55.09	51.09
Body mass index, kg/m^2^	21.76 ± 2.47	23.09 ± 2.76	24.43 ± 3.03	25.52 ± 3.15	26.65 ± 3.17	27.29 ± 2.96
< 25, %	69.74	49.88	32.26	20.54	11.64	2.14
≥ 25, %	30.26	50.12	67.74	79.46	88.36	97.86
Waist circumference, cm	73.68 ± 7.12	78.09 ± 7.67	82.32 ± 8.12	85.52 ± 8.26	89.04 ± 7.85	93.08 ± 5.53
Total cholesterol, mg/dl	185.13 ± 30.92	191.59 ± 34.31	201.36 ± 37.03	206.72 ± 39.52	208.44 ± 41.61	208.33 ± 42.91
HDL cholesterol, mg/dl	62.18 ± 13.71	56.42 ± 16.63	52.54 ± 16.99	49.5 ± 16.32	47.63 ± 15.68	46.3 ± 14.96
LDL cholesterol, mg/dl	106.98 ± 28.91	113.5 ± 31	118.65 ± 34.39	119.9 ± 37.52	119.48 ± 39.8	118.78 ± 41.44
Triglycerides, mg/dl^a^	74.86 (74.84–74.88)	98.33 (98.3–98.37)	135.17 (135.1–135.24)	169.91 (169.79–170.02)	189.52 (189.34–189.7)	197.51 (197.11–197.92)
Systolic blood pressure, mmHg	111.55 ± 9.28	121.12 ± 13.75	126.45 ± 14.39	130.58 ± 14.32	133.66 ± 14.07	135.36 ± 14.15
Diastolic blood pressure, mmHg	69.95 ± 7.28	75.65 ± 9.47	78.83 ± 9.82	81.18 ± 9.89	82.75 ± 9.86	83.46 ± 9.93
Fasting plasma glucose, mmol/l	86.76 ± 7.62	91.55 ± 10.41	95.39 ± 11.5	97.85 ± 11.76	101.95 ± 11.35	108.78 ± 6.96
Current smokers	1,317,896 (21.02)	1,454,996 (26.1)	1,124,501 (28.78)	671,147 (28.09)	275,492 (25.43)	55,468 (22.1)
Heavy alcohol drinker (≥ 30 g/day)	254,425 (4.06)	355,292 (6.37)	323,275 (8.27)	216,731 (9.07)	96,259 (8.89)	21,012 (8.37)
Regular exercise (yes)	3,204,800 (51.11)	2,829,184 (50.75)	1,957,155 (50.09)	1,170,131 (48.98)	513,768 (47.43)	112,812 (44.95)
Income (lower 25%)	1,755,411 (28)	1,514,204 (27.16)	1,003,101 (25.67)	597,012 (24.99)	268,774 (24.81)	62,445 (24.88)

Data are presented as mean ± standard deviation (SD) or proportions (%)

^a^Geometric means (95% CI) calculated using Wald method for means

### Association between the number of metabolic syndrome components at baseline and diabetes risk during follow-up

During an average 5.13 years of follow-up, 1,906,963 subjects were diagnosed with type 2 diabetes. Table 2 shows the incidence rates and HRs of type 2 diabetes according to the number of metabolic syndrome components at baseline. The age- and sex-adjusted incidence rates of developing diabetes were 10.86, 15.53, 21.35, 29.14, 38.69 and 56.65 per 1000 person-years for participants with zero through five components at baseline, respectively. After adjusting for age, sex, alcohol consumption, smoking status, exercise, income, and BMI, the multivariable-adjusted HRs for developing diabetes were 1.401 (95% CI 1.394–1.409), 1.862 (95% CI 1.852–1.872), 2.47 (95% CI 2.456–2.484), 3.164 (95% CI 3.144–3.184) and 4.501 (95% CI 4.466–4.537) for participants with one to five components at baseline, respectively, compared to those with no components (Table 2).

**Table 2:** Multivariable-adjusted hazard ratios for developing diabetes according to the number of metabolic syndrome components

	Number of components						Trend P
	0	1	2	3	4	5	
Cases, n	218,603	385,479	455,944	426,612	303,566	116,759	
Follow-up duration (person-years)	31,043,596.21	28,642,155.32	20,577,529.59	12,419,916.48	5,887,174.59	1,415,272.2	
Crude incidence rate (per 1000 person-years)	7.0418	13.4584	22.1574	34.349	51.564	82.4993	
Age- and sex-adjusted incidence rate (per 1000 person-years)	10.8634	15.5303	21.3481	29.1435	38.6941	56.6539	
Adjusted HR^a^ (95% CI)	1 (ref.)	1.401 (1.394,1.409)	1.862 (1.852,1.872)	2.47 (2.456,2.484)	3.164 (3.144,3.184)	4.501 (4.466,4.537)	< 0.0001

^a^Adjusted for age, sex, alcohol consumption, smoking status, exercise, income, and BMI

### Diabetes risk according to the number of metabolic syndrome components stratified by age, sex, and BMI

**Fig. 1:**
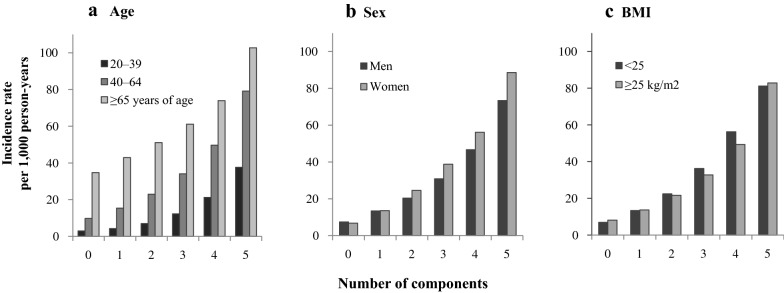
Incidence rates of type 2 diabetes according to the number of metabolic syndrome components by age, sex, and BMI. Incidence rates **a** by age, **b** by sex, **c** by BMI. There was a significant increase in diabetes incidence with numbers of metabolic syndrome components was significant (P < 0.0001)

The incidence rates of diabetes according to the number of metabolic syndrome components after stratification of study participants into groups based on age (20–39, 40–64, ≥ 65), sex (men vs. women), and BMI (below or above 25) are shown in Fig. 1. The incidence rates of diabetes were 34.72, 42.91, 51.07, 61.14, 73.93, and 102.76 per 1000 person-years for participants ≥ 65 years; 9.73, 15.35, 22.94, 34.07, 49.69, and 79.19 for participants 40–64 years; and 2.96, 4.23, 6.99, 12.28, 21.23, and 37.6 for participants 20–39 years of age, respectively. The incidence rates among women were higher than those among men with two or more components. There was no difference in incidence rates according to the number of metabolic syndrome components stratified by BMI.

**Fig. 2:**
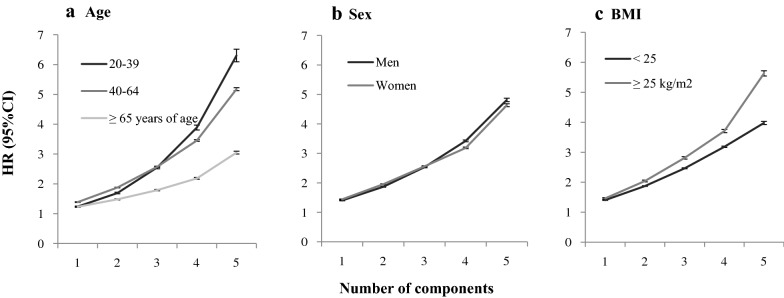
Hazard ratios (95% CI) of type 2 diabetes according to the number of metabolic syndrome components by age, sex, and BMI. Hazard ratios **a** by age, **b** by sex, and **c** by BMI. The interaction between metabolic syndrome components and subgroups (age, sex, and BMI) is statistically significant (P < 0.0001). There was a significant increase in diabetes risk with numbers of metabolic syndrome components (P < 0.0001). Multivariable hazard ratios were adjusted for age, sex, alcohol consumption, smoking status, exercise, income, and BMI. Error bars represent upper 95% CI

Figure 2 shows the risk of developing diabetes according to the number of metabolic syndrome components among individuals stratified by age, sex, and BMI. The interaction between metabolic syndrome components and subgroups (age, sex, and BMI) is statistically significant (P < 0.0001). Multivariable-adjusted hazard ratios for incident diabetes among individuals with one through five components at baseline were 1.23, 1.48, 1.79, 2.18, and 3.05 for participants ≥ 65 years; 1.39, 1.88, 2.57, 3.45, and 5.18 for participants 40–64 years; and 1.24, 1.69, 2.55, 3.89, and 6.31 for participants 20–39 years of age, respectively, compared to those with no components. The risks of diabetes were the highest in the 40–64 year age group among participants with one or two components and in the 20–39 year age group among those with four or five components. Diabetes risk was the lowest in the ≥ 65-year age group for all numbers of components.

There was no difference in diabetes risk according to the number of metabolic syndrome components between men and women. The risk of diabetes across the number of metabolic syndrome components was greater in participants with a BMI ≥ 25 than in those with a BMI < 25. Multivariable-adjusted hazard ratios for incident diabetes in individuals with five components were 5.63 for participants with a BMI ≥ 25 and 3.98 for those with a BMI < 25.

## Discussion

In this large-scale prospective cohort study of 19.5 million adult Korean participants, we investigated the association between diabetes risk and the number of metabolic syndrome components by age, sex, and BMI. The number of metabolic syndrome components at baseline was significantly associated with an increased risk of diabetes over a 5-year period, independent of sociodemographic characteristics. The risk of diabetes was more strongly associated with the number of metabolic syndrome components among younger adults. In addition, the risk of diabetes across the number of metabolic syndrome components was greater in individuals with a BMI ≥ 25.

Our findings suggest that the number of metabolic syndrome components provides additional value for predicting the development of diabetes. Several studies have confirmed that metabolic syndrome, regardless of its definition, is a significant predictor of diabetes in various populations [20]. Previous studies have also shown that diabetes risk increases with the number of metabolic syndrome components [6–8]. In the present study, diabetes risk increased with the number of metabolic syndrome components. The presence of only one metabolic syndrome component was significantly associated with diabetes risk compared to the presence of no components.

We prospectively examined the risk of diabetes according to the number of metabolic syndrome components by age, sex, and BMI. There was a significant increase in diabetes incidence with increasing numbers of metabolic syndrome components. When participants were divided into three age groups (20–39, 40–64, ≥ 65 years), the incidence rates of diabetes were highest for individuals ≥ 65 years old and were lowest for participants 20–39 years old across all numbers of components. Diabetes risk was more strongly associated with the number of metabolic syndrome components among younger individuals. There was no significant difference in the incidence rate or risk of diabetes between men and women.

As expected, the number of metabolic syndrome components was positively correlated with age. The incidence rates of diabetes were greater in older adults; however, the risk of diabetes by number of metabolic syndrome components was higher in young adults. Among participants with five components, the risk of incident diabetes was 6.31-times higher for people 20–39 years of age, 5.18-times higher for participants 40–64 years, and 3.45-times higher for participants ≥ 65 years than for those without metabolic syndrome components. These findings suggest that the number of metabolic syndrome components is important, particularly for young adults with metabolic syndrome. As the prevalence of type 2 diabetes in the young adult population is dramatically increasing [21, 22], it is important to identify individuals who have a high risk of developing type 2 diabetes in this age group.

The major predisposing risk factors of type 2 diabetes among young adults are obesity, family history, and sedentary lifestyle factors such as physical activity, diet, smoking, and alcohol consumption [23, 24]. A study revealed that obesity, family history, hypertension and dyslipidaemia were independent risk factors for early-onset type 2 diabetes [25]. These risk factors are similar to those associated with later-onset type 2 diabetes [26]. Early-onset type 2 diabetes leads to a longer lifetime exposure to hyperglycaemia and consequently more severe long-term complications [27]. Additionally, the course of early-onset type 2 diabetes could be more rapid and disruptive than that of older-onset type 2 diabetes, leading to early morbidity and poor quality of life [28]. Therefore, it is warranted to apply early lifestyle interventions to prevent the development of type 2 diabetes in young adults with metabolic syndrome components.

BMI is strongly and independently associated with the risk of type 2 diabetes [29, 30]. We evaluated the risk of diabetes across the number of metabolic syndrome components by BMI status. We observed that the risk of diabetes across the number of metabolic syndrome components was greater in individuals who were overweight or obese than in those who had normal BMIs. Moreover, we examined the association between the number of metabolic syndrome components and diabetes risk after adjustment for BMI. The number of metabolic syndrome components was associated with an increased risk of diabetes after adjustment for traditional risk factors such as age, sex, alcohol consumption, smoking status, exercise, income, and BMI. The use of waist circumference to assess abdominal adiposity is superior to that of BMI. WC is a better predictor of metabolic syndrome than BMI and is widely used in the definition of metabolic syndrome [17, 31]. WC is more strongly associated with an increased risk of type 2 diabetes and cardiovascular disease than BMI [32, 33]. In this study, after adjusting for risk factors, including BMI, the association remained significant. This association was independent of the risk predicted by increased BMI. Our findings showed an independent correlation of WC, but not BMI, with obesity-related diabetes risk. We should screen for WC as well as BMI since the early detection of individuals with metabolic abnormalities may be beneficial in the prevention of diabetes.

The strengths of our study include its longitudinal population-based design, a sufficient number of type 2 diabetes events, a high follow-up rate, and a nationally representative data set. However, the present study has some limitations that could be addressed by further investigation. First, the diagnosis of metabolic syndrome was based on a single measurement at baseline, similar to the method used in other epidemiological studies. The number of metabolic syndrome components of individuals may have changed during the follow-up period because of lifestyle factors and medications; therefore, risk estimates may also have changed. Second, because type 2 diabetes was defined based on the prescription of anti-diabetes drugs and the presence of relevant ICD-10 codes, type 2 diabetes patients who were not diagnosed prior to the analysis might have been misclassified as not having diabetes. Third, although we adjusted for potential confounding factors, we could not completely exclude reverse causality or the effects of unmeasured confounding factors. Finally, the data represent outcomes of South Korean citizens, a homogenous ethnic population, and this study population may be considered a limitation in regard to generalizability. Further large-scale prospective studies are required to verify these results in other populations.

## Conclusion

Our stratified analysis indicated that younger adults had a higher risk of diabetes across the number of metabolic syndrome components than older adults. These results imply that early prevention and intervention of metabolic syndrome components particularly in young adults are important. In addition, the risk of diabetes according to the number of metabolic syndrome components was greater in obese individuals. Our findings indicate that age and BMI-specific risk stratification by the number of metabolic syndrome components can identify individuals with a high risk of developing diabetes.

## Data Availability

The data that support the findings of this study are available from the National Health Insurance Service but restrictions apply to the availability of these data, which were used under license for the current study, and so are not publicly available. Access to the dataset can be obtained through the Health Insurance Data Service home page (http://nhiss.nhis.or.kr).

## Abbreviations

NHIS: National Health Insurance Service
NCEP ATP III: National Cholesterol Education Program Adult Treatment Panel III
WC: waist circumference
HDL: high density lipoprotein
BP: blood pressure
BMI: body mass index
FPG: fasting plasma glucose
TC: total cholesterol
LDL: low density lipoprotein
